# Callendula-Officinalis*Sources of information: John W. Clark, M. D., *Homœopathic World*, ’90; “C. W.” in *Homœopathic World*, ’90; Robert T. Cooper, M. D., Homœopathic Review, ’89, and *Homœopathic World*, ’92; Malcolm Macfarlan, M. D., Homœpathic Physician, xii, 276; from Petroz by Agricola, *Homœopathic World*, ’92.

**Published:** 1893-06

**Authors:** W. A. Yingling

**Affiliations:** Nonchalanta, Kansas


### CALENDULA-OFF.*

W. A. Yingling, M. D., Nonchalanta, Kansas.

Mind : Extremely nervous, starts at sudden noises ; fretful. A feeling as of some overwhelming calamity impending ; almost unbearable.

Head : Moves head up and down ; headache, piercing ; splitting ; tearing ; rending in sides ; vertex ; occiput ; lancings in the forehead ; tingling in the vertex ; weight on the brain ; paralysis after apoplexy ; congestion ; constriction.

Eyes : Rolls eyes ; fixed (in convulsions) ; dark rings around eyes ; an appearance as of skin over inner section of left eye ;

---

* Sources of information : John W. Clark, M. D., *Homœopathic World*, '90 ; "C. W." in *Homœopathic World*, '90 ; Robert T. Cooper, M. D., *Homœopathic Review*, '89, and *Homœopathic World*, '92 ; Malcolm Macfarlan, M. D., Homœpathic Physician, xii, 276 ; from Petroz by Agricola, *Homœopathic World*, '92.

traumatic ophthalmia; painful because of the headache; puffy under the eyes; tetters, shootings, pain, inflammation; eyelids adherent; sclerotic blue; spots (*taches*) on the globes; diplopia; sees only half of objects; weakness of the sight; movement before the eyes; a thick veil before the eyes.

EARS: Hearing acute; starts at noises.

"* * * the deafness had come on after bathing: both membranes normal in appearance, there was no discharge whatever, and there was an inability to distinguish with the left ear from whence sounds were coming, and which was always worse in damp weather." [Prescription of *Calendula* in the form of snuff made from the Ø and Sacch.-lac.]—Dr. Robert T. Cooper, *Homœopathic World.*

Hears best on a train or in a busy thoroughfare.

Hearing is worse when she takes cold, which she always does in damp weather; worse when fatigued.

Cannot hear two persons speaking together. Hears church bells and distant sounds fairly.

Deafness worse in *damp surroundings.*

Deafness where eczematous conditions are present.

"Moist catarrhal manifestations of vascular deafness."

Aching of left internal ear; discharge from the ears; noises (bruit). Murmur in the head; hearing affected by music; sensation of heat in ears; hearing affected, with cold feet.

NOSE: Cold in head with thick green discharge; severe sneezing; tetters reaching to temples; cancer; sensibility.

Coryza in general; in one nostril; with excretion clear; yellow.

FACE: Puffy and swollen, particularly under the eyes.

TONGUE, TASTE, ETC.: Blisters on tongue; bitter taste of food; taste of pepper; watery; mucous; putrid; after taste of aliments.

MOUTH: Dark circle round mouth in convulsions; acid fluids in mouth; mucosities in mouth; reddish scum in mouth; saliva acrid; inflammation; excrescences; hemorrhage of; opens mouth with difficulty; burning heat in œsophagus; swallowing difficult; chronic hoarseness.

APPETITE: Hunger immediately after nursing; constantly wants the breast.

Bulimy with prompt satiety; desire for beer; for vegetables; hunger at midday; hunger immoderate in the evening.

Hunger, thrist, and flow of urine; malaise after fatty foods; sense of satiety; absence of thirst.

NAUSEA AND VOMITING: Vomiting milk, curdled, slimy; thick sticky mucus.

Eructations; hiccough; pyrosis.

*Heartburn with horripilations.*

Risings; sweetish, rancid; food lies heavy; nausea in precordial region; nausea alternating with diarrhœa; nausea in the *chest.*

Vomitings of bitter matter; vomitings of black matter; vomitings with heat, pale face; vomitings "in spittings;" vomitings periodic; vomitings after drinking fluids; vomitings, convulsions during.

STOMACH: Hiccough, violent, persistent; worse after nursing, and after vomiting.

Pain after nursing; throbbings; contraction; cutting pains; *sinking* of cardia; sensation of rupture of cardia; swelling in precordial region; sensation of ulceration; sense of emptiness; *lancinating pain in splenic region;* unable to support clothing.

ABDOMEN: Umbilical hernia, after much straining at stool and screaming.

Epigastric *distention.*

Cuttings in sides; in region of kidneys.

Tearing in inguinal region.

Senses of tension, tumefaction. Contraction.

STOOLS AND ANUS, ETC.: Constipation and flatulence.

Makes a grunting noise as if passing stool, but only passes wind [in a child].

Stool deep reddish-yellow-marigold color, chopped appearance, at times frothy, strong odor.

Much straining. Stool expelled forcibly to a distance if the diaper is not on. Anus excoriated by stool. Diarrhœa during dentition. Diarrhœa day and night. Chronic hardness of stools.

Anus, smartings of; pressure in; hemorrhage from; constriction. Herpatic eruptions on anus and perineum. Hemorrhoids, burning. Pain, weight in rectum. Dysentery with gastric affections.

URINE: The difficult urination common to old people.

Dark, offensive, staining the diaper deeply. Greatly diminished.

Distressing urging to urinate. Distressing urging to urinate in pregnant women.

Brown sediment. Suppression of urine. Urine deep colored. Urine frequent. Involuntary. Painful urination. Vesical cuttings. Enuresis nocturnal. Frequent inclination and drinking. Frequent emissions of pale, clear, hot, and even burning urine. Lacerating in the urethra during the chilliness.

FEMALE SEXUAL ORGANS: *Warts on the mouth of the uterus.* Pale blood flows from the uterus. *Menses suppressed.* Leucorrhœa. Pain in back of women. Cough during suppressed menses.

COUGH: In open air. After walking. Mid-day. In breathing. During suppression of *menses. From roughness of chest. With expectoration of globular sputa. With expectoration green, tasting badly.*

*With distention of the inguinal ring.* With hoarseness, malaise, heat of face.

HEART, PULSE: Palpitations. Pulse full.

CHEST: Sensations of dilatation; constriction; pressure. Blue around clavicles. Sensibility in mammary glands. Ulcers, burnings in. Swellings of mammæ with or without pain.

NECK AND BACK: Eruption on neck. Pains in region of kidneys.

UPPER EXTREMITIES: Arms and hands twitch.

Heat, burning in the arms. Cramps. Pain, paralytic, at shoulder articulation. Paralysis of forearm. Stiffness, arthritic, of the fingers. Ulcers on the fingers. Periostitis. Acute pain in ball of left thumb.

LOWER EXTREMITIES: Draws up the legs. Stiffness in feet articulations. Fetid sweats of the feet. Severe pain in arch of right foot.

LIMBS IN GENERAL: Fractures.

Rheumatic drawing pains only during motion; restless nights.

FEVER, CHILLS: Shuddered after vomiting. Sensitive to the open air. Sweat stains the linen. Sweat with horripilation; nocturnal; weakening. Horripilations.

Heat in the afternoon, with frequent thirst; chilliness and shiverings intermixed, *particularly after drinking.*

Heat in the evening with coldness of head and hands, intermingled with shivering, with aversion to drinks.

SLEEP: Restless. Restless four to five A. M.

Starts in sleep. Gasping in sleep. Wakes up screaming.

Feels as if he would fall from a height when dropping asleep.

Attempts to uncover. Drowsiness with ill-humor and delirium. Restless nights. Constant waking.

NERVES: Screams. Hands and arms twitch. Convulsions eleven A. M., immediately after nursing. Extremely nervous; starts at noises. Great tendency to start and twitch with nervousness. Convulsions during vomiting and colic.

TISSUES: Jaundice. Does not lose flesh in spite of vomiting. Bloody and serous infiltrations of cellular tissue in open wounds and ulcers. Destructive syphilitic ulcers.

Submaxillary glands are painful to touch. Painful axillary glands. Incised or lacerated wounds.

It limits suppuration and assists in securing primary union along incised surfaces.

Suitable in cases with loss of soft parts.

[I took a case of great loss of soft parts from the bite of a stallion, where Bichloride of Mercury, strong, had been used to excess. After days there was no repair of parts, with profuse suppuration. I thoroughly cleansed parts and applied diluted Calendula in water, keeping parts moist with it. Within twenty-four hours from the use of Calendula, the repair was noticeable to the unprofessional eye, suppuration remained at the minimum, with a good recovery.—W. A. Y.]

SKIN : decided yellow ; jaundice ; suggillations.

Promotes favorable cicatrization, with least amount of suppuration.

Horripilations ; semi-lateral ; *on being touched ; goose-flesh.*

" * * * the ulcer was larger in size than a one rupee silver coin, covered by a yellowish crust, formed by drying up of the discharges from it. When the crust was taken off, I noticed much slough all over, proud flesh in the centre, with depression between the proud flesh and the raised edges and fissure-like cracks all over the ulcer, with much painful swelling of the leg and foot and redness of the surrounding parts, looking like diffused erysipelatous inflammation. * * * prescribed *Calendula* Φ lotion. * * * Slough, proud flesh, and raised edges were the indications for my prescribing *Calendula*."—R. K. Ghosh, M. D., in *Homœopathic World.* [A lotion of the 30th or 200th will be found to be as efficacious as that of the tincture, with the higher potencies internally.—W. A. Y.]

GENERALITIES : Symptoms intermit.

*Great disposition to take cold, especially in damp weather.*

Wounds that suppurate profusely ; incised or *lacerated* wounds ; bloody and serous infiltration of open wounds and ulcers ; burns, concussion from blows, shock, fall, etc. ; pains, lancinations, excoriations ; lassitude, difficult movement ; tension of the body ; pains, stabbing from within to without ; pains, of excoriation to the touch ; *mechanical lesions ;* blood clotted ; delirium from pain ; sensibility.

*Aggravation in damp, cloudy weather.*

TIME : Four and five A. M., restless.

Eleven A. M., convulsions.

Five P. M., vomiting.

Evening and night, stools, restless.

RELATIONSHIPS : Antidote, *Chelidonium ; Sang.* helped the catarrhal state ; *Æthusa* controlled the vomiting; *Cham.* the fretfulness; *Lyc.* was of some assistance ; probably *Rheum* is antidotal.

